# Vagueness as an implicit-encoding persuasive strategy: an experimental approach

**DOI:** 10.1007/s10339-023-01171-z

**Published:** 2024-01-29

**Authors:** Giorgia Mannaioli, Alessandro Ansani, Claudia Coppola, Edoardo Lombardi Vallauri

**Affiliations:** 1 Department of Philosophy, Communication, and Performing Arts, Roma Tre University, Rome, Italy; 2 Department of Music, Art, and Culture Studies, University of Jyväskylä, Jyväskylä, Finland; 3 Department of Foreign Languages, Literatures, and Cultures, Roma Tre University, Rome, Italy

**Keywords:** Vagueness, Implicit persuasion strategies, Shallow processing, Self-paced reading experiment

## Abstract

The paper provides novel theoretical and experimental perspectives on the functioning of linguistic vagueness as an implicit persuasive strategy. It presents an operative definition of pragmatically marked vagueness, referring to vague expressions whose interpretation is not retrievable by recipients. The phenomenon is illustrated via numerous examples of its use in predominantly persuasive texts (i.e., advertising and political propaganda) in different languages. The psycholinguistic functioning of vague expressions is then illustrated by the results of a self-paced reading task experiment. Data showing shorter reading times associated with markedly vague expressions as compared to expressions that are either (a) lexically more precise or (b) made precise by the context suggest that the former are interpreted in a shallow way, without searching for and/or retrieving exact referents. These results support the validity of a differentiation between context-supported vs. non-supported vague expressions. Furthermore, validation of using marked vagueness as a persuasive implicit strategy which reduces epistemic vigilance is provided.

## Introduction

The present work is part of a larger research project on linguistic implicit-encoding strategies (presuppositions, implicatures, topicalizations, and vague expressions) considered as potentially manipulative strategies (Lombardi Vallauri 2016, 2019a; Lombardi Vallauri et al. 2020; Lombardi Vallauri and Masia 2014). The present study focuses on linguistic vagueness. The purpose of this study is (i) to provide new grounds for the theoretical discussion of vagueness as an implicit strategy; (ii) to provide a pragmatic analysis of vagueness in persuasive texts, such as contemporary political propaganda and commercial advertising; and (iii) to provide new experimental data about the reading behavior of vague expressions. The work is structured as follows. In "Vagueness as an implicit strategy: a pragmatic definition" section, we present a characterization of vagueness to distinguish between a more “physiological” level of vagueness and a more pragmatically “marked” one, and we suggest that cases of the latter may function as a persuasive implicit strategy. In "A pervasive strategy in persuasive texts" section, we discuss examples of the use of marked vagueness in persuasive texts from different languages. "The persuasive potential of vague expressions: diverting epistemic vigilance and shielding from responsibility" section suggests that vagueness should be considered a persuasive device on cognitive grounds. In "Experimental application" section, we present experimental data on the processing costs of different levels of vagueness, as measured by reading times. In "Discussion" section, conclusions are drawn as well as future perspectives provided.

### Vagueness as an implicit strategy: a pragmatic definition

Over the last decades, it has become increasingly acknowledged that vagueness intended as meaning underspecification is a characterizing feature of verbal languages, arguably regarding every type of linguistic expression (Bonini et al. 1999; Crystal et al. 1975; Evison et al. 2007; Lakoff 1972; O’Keeffe and Cheng 2015; Russell 1923). A “physiological” level of vagueness, referring to the fact that signs are intrinsically underspecified, exact references inferred and pragmatically adjusted on a contextual basis, is intrinsic to the linguistic code and arguably represents the default condition in everyday communication (Simone 2005; Wilson and Carston 2007; Lombardi Vallauri 2019b). However, considering vagueness as extension indeterminacy, it is generally acknowledged that certain expressions are typically vaguer than others. For instance, the predicate *tall* is considered vaguer than the predicate *Swedish*, because it happens more frequently that one cannot say what a speaker means *exactly* by saying *tall* compared to when someone says *Swedish*.

In the present study, we provide an analysis of vague expressions, focusing on the precising role of context, showing there is a substantial semantic, rhetorical (concerning persuasion), and psycholinguistic difference between context-supported and context-unsupported vague expressions. For example, the vague predicate *tall*, considered outside of context, as an item in the language system, has an imprecise extension. However, it can be precised at different levels within different contexts, up to becoming semantically determinate. To illustrate gradations of precisation within contexts at the utterance level, we provide the following classifications, (1)–(4), with (1) describing an *extremely indefinite extension*, and (4) a *definite extension*:

1. I love *tall* mountains.
2. *Tall* animals have an evolutionary advantage in the savannah.
3. They want a *tall* guy to play the superhero role.
4. [Two pieces of furniture: 120cm and 190cm] I’ll buy the *tall* one.

As can be seen, in (1) the extension of *tall* remains very vague. In (2), it becomes somewhat less vague, as the maximum height for a savannah animal is about 5–6 m (which is an adult giraffe’s height). In (3), the extension of *tall* can be calculated as approximately being between 175 and 200 cm of height. In (4), *tall* comes to mean exactly the object *x’*s height. The different degrees of context precisation may be seen along a continuum, which goes from extremely indefinite to precised. We assume that in cases such as (3) and (4), the intrinsic vagueness of *tall* is not even noticed by interlocutors, as the recipient is able to form a good-enough mental representation of the speaker’s meaning (Ferreira et al. 2002). For the present work, we will focus on cases in which linguistic expressions’ reference/extension remains vague despite the integration of context information, i.e., cases in which the context does not have a precising function (Channell 1994; Peirce 1902). Contextually non-precised vague expressions (i.e., vague expressions that are not made precise by their context) allow recipients to form numerous different mental representations. An example is provided in (5):

5. Now I have to take care of *some stuff*, I’ll call you later

As can be seen, the expression *some stuff* in (5) does not enable the recipient to build a clear mental representation of what the speaker is referring to.

For the present work, we will propose a pragmatic analysis of these cases of context-unsupported vagueness, which we see as a more “marked” level of vagueness, and that we consider as an implicit-encoding strategy (see also Brown and Levinson 1987; Cotterill 2007; Jucker et al. 2003; Lombardi Vallauri 2016, 2019b; Overstreet and Yule 1997b, 1997a). Thus, we consider as *marked vagueness*:

Expressions through which the speaker conveys contents whose extension is underdetermined, part of those contents remaining implicit and not univocally retrievable. Such expressions are therefore neither completely verifiable nor falsifiable.

Specifically, we will explore cases in which markedly vague expressions’ use is persuasive and potentially manipulative (i.e., non-fully cooperative), due to its power to implicitly convey non-*bona fide* true contents^1^ (Lombardi Vallauri 2016, 2019b).

We assume that conveying contents in an underspecified way, such as through semantically underdetermined expressions, is cooperative when omitting irrelevant, contextually retrievable, or unavailable information. In contrast, we consider vagueness as a non-fully cooperative and potentially manipulative implicit strategy when it does not overtly express information available to the speaker, relevant in the ongoing discourse, and not retrievable from the context. This leaves the receiver uncertain about what exactly the speaker has in mind.^2^

**Fig.1:**
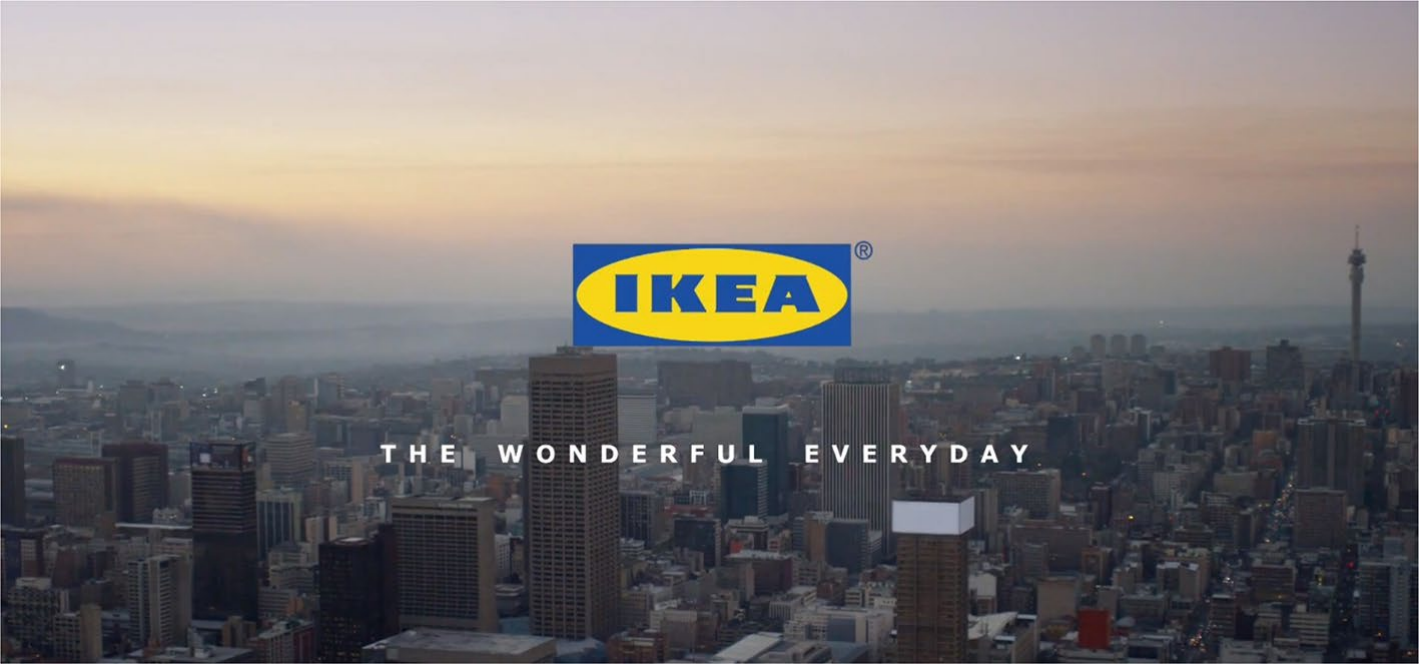
Ikea 1

**Fig. 2:**
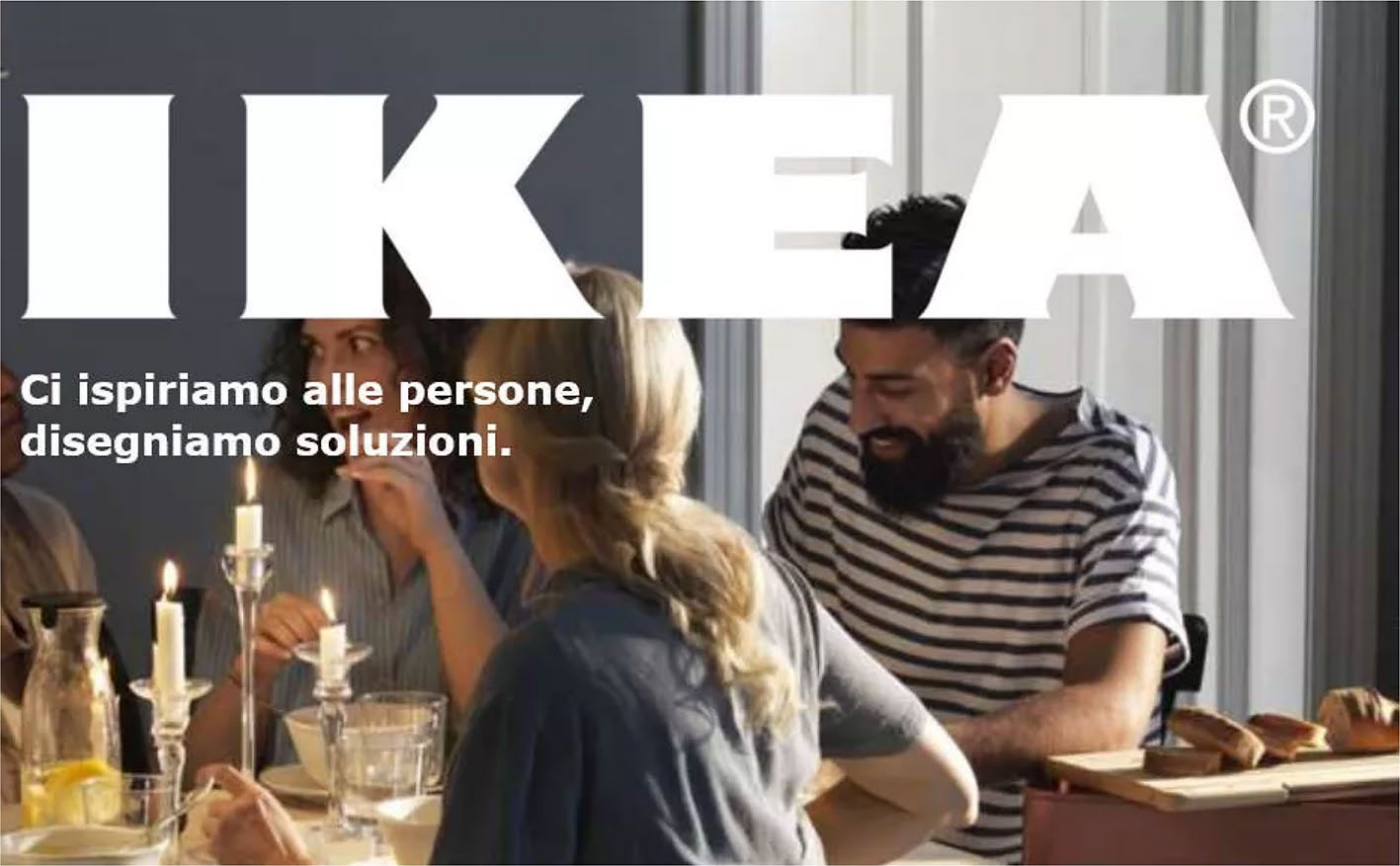
Ikea 2

More in detail, we suggest that marked vagueness can be used in a persuasive and potentially manipulative way when including questionable contents in its implicit potential meanings. When a vague expression is used in a real text, the receiver can typically construct a salient and plausible mental representation of it based on contextual hints. In the case of marked vagueness, however, as vague expressions lack contextual precision, addressees may associate them with a wide range of representations (some of which may even be false or questionable), depending on their personal inclinations. Let us see two examples in (6) and (7)^3^ (Figs. 1, 2):

6. *IKEA. The wonderful everyday*.
7. *IKEA. Ci ispiriamo alle persone, disegniamo soluzioni.* (IKEA. We are inspired by people, we design solutions).^4^

Both Ikea’s slogans employ denotatively fuzzy and generic expressions (e.g., the nominalized adjective *wonderful,* and the noun *solutions*, respectively), whose precise extension is difficult or impossible to retrieve. In (6), it is unclear which wonderful characteristics consumers should expect: high quality of the furniture? Good prices? Nice design? Importantly, some of the different possible precisifications of the vague expression may be questionable or false. Similarly, in (7) the noun *people* refers to humans in general, so *to be inspired by people* is also very generic, just as *we design solutions* (different people may think of different kinds of problems and solutions). The persuasive potential of these underdetermined expressions rests on the fact that they allow the source to convey a message that is appealing to very different receivers, i.e., to customers that prioritize different things (e.g., either some price range, or product quality some of which may not be true of the brand). Through the vague message, the advertiser potentially hints at all of them, but does not explicitly commit to any precise content. Increased precision would limit the text’s appeal to only a subset of potential customers. This would reduce its attractiveness to others, and at the same time, make it easier for them to identify false content.

The assumption that vagueness is an effective persuasion device seems to be further confirmed by the pervasive use of vague expressions in typically persuasive text types, such as advertising and political propaganda (Lombardi Vallauri 2019a; Lombardi Vallauri et al. 2020). In the following section, we will argue that the semantic combination of fuzzy denotation and clear connotation is what maximizes the persuasive impact of vague messages.

### A pervasive strategy in persuasive texts

#### Advertising

**Fig.3:**
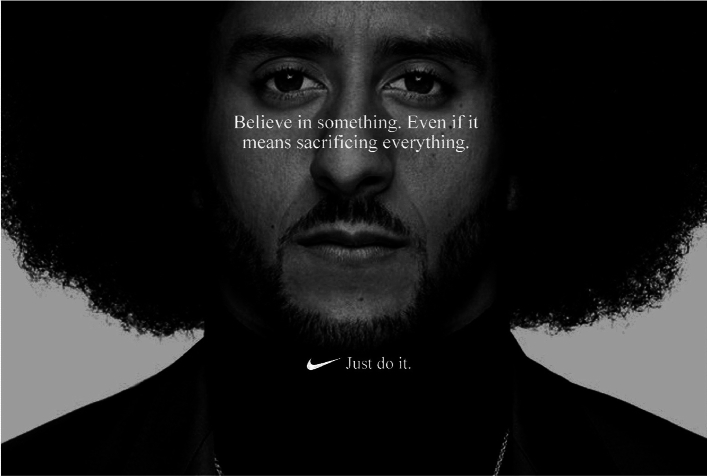
Nike

Advertisers seem well aware of the persuasive potential of vagueness, which often takes up most of the message (Fig. 3).

8. * Believe in* ***something****. Even if it means sacrificing ****everything****. Just ****do it***.

In Nike’s advertisement in (8), the indefinite pronouns *something*, *everything*, the personal pronoun *it* and the verb *do* are intrinsically underspecified, possibly referring to any kind of states, events, and referents. As Cutting observes on the discourse of advertising:

“Advertising employs ‘do’ and ‘happen’ and ‘it’ in promotional slogans to include everything, everyone and every action, and impose assumptions of relevance on all who read or hear it. [...] These slogans are expressed in unspecific terms in order to reach all potential consumers by making each believe that the company or bank can satisfy their individual personal needs” (2007:232)

**Fig. 4:**
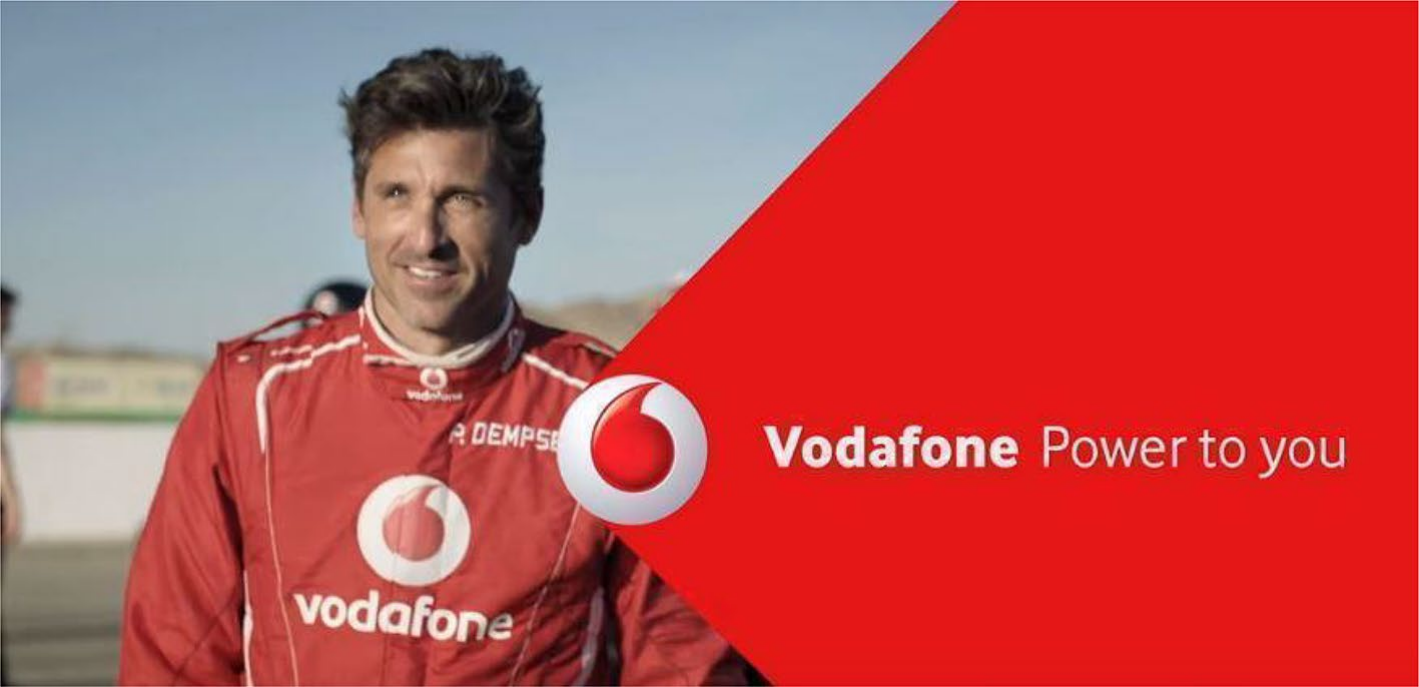
Vodafone

The persuasive potential of slogans like the one in (8) lies specifically in the fact that every single receiver can interpret vague expressions as referring to anything they want, subjectively interpreting the message according to their own personal taste, despite the advertiser not committing to anything in particular (poor denotation and clear connotation, see "Vagueness as an implicit strategy: a pragmatic definition" section; Fig. 4).

In this advertisement, the phone operator also strategically exploits vagueness by promising “power” to its clients. The connotation is overall very positive, but what exactly does “power” entail? Good reception? Limitless internet data? Wider choice of phone plans? Readers are asked to imagine all kinds of positive referents according to their own ideas and needs. Among these, some may be beyond what the phone operator can deliver, and they cannot be held accountable if consumers’ expectations are not met.

**Fig. 5:**
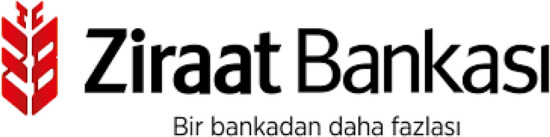
Ziraat Bankasi

**Fig. 6:**
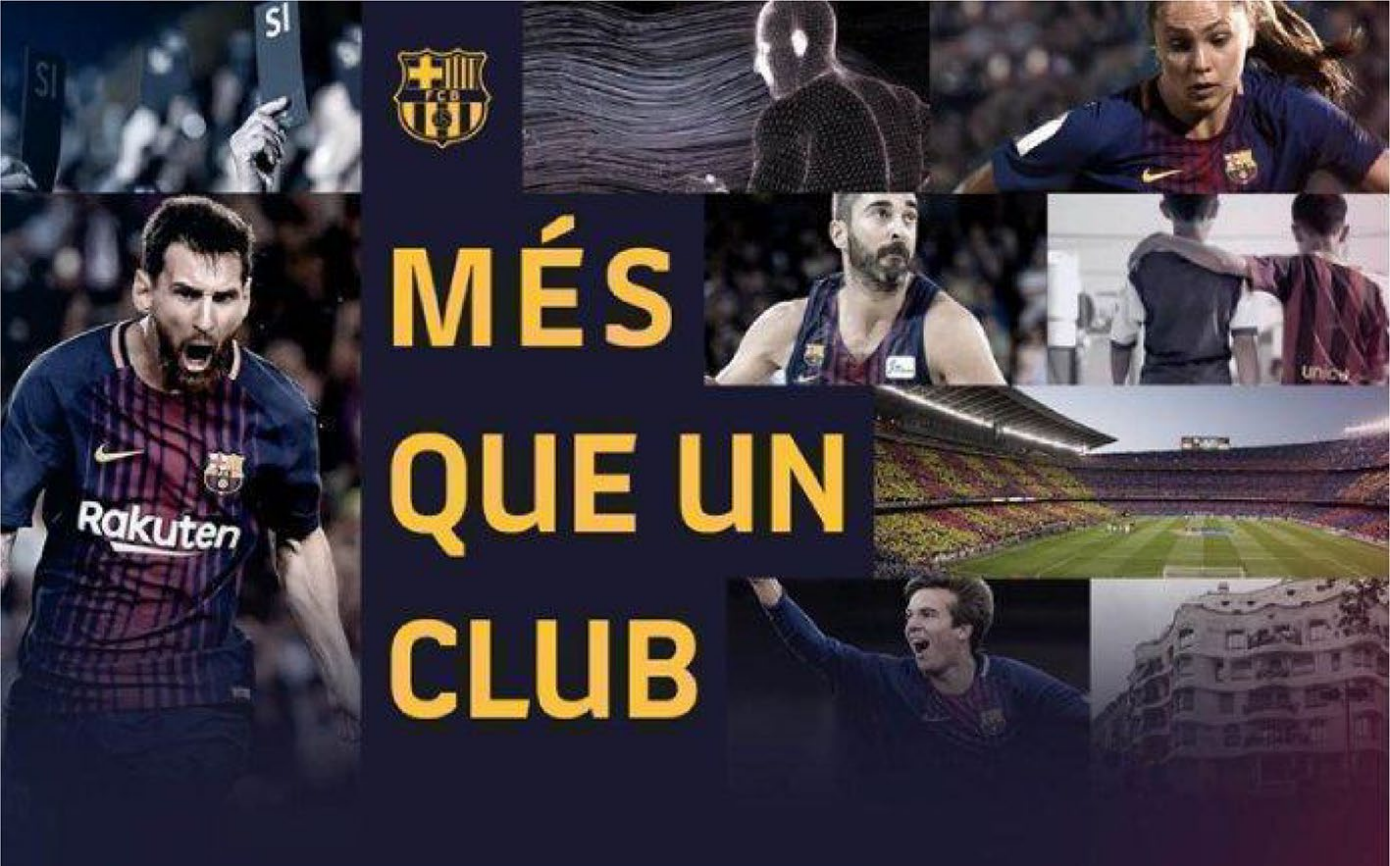
Més que un club

A semantically vague construction that interestingly is exploited by advertising in several languages and in different cultural environments is the (*much) more than* construction (Fig. 5):

(5) (Turkish) *Ziraat Bankası. Bir bankadan daha fazlası.* (Ziraat Bank. Much more than a bank) (Fig. 6)

(7) (Catalan) *Més que un club.* (More than a club).

Vague adverbial structures of quantification, such as *much more,* instantiate a comparison with competitor products by saying that the promoted brand has *something more* than others; yet, they leave unsaid which is the exact surplus offered by the promoted brand, i.e., the quality which is predicated; and to what extent they can offer (*much) more* of it. Such a strategy significantly enhances the source’s manipulative potential, as it can be employed also in cases in which the promoted product has actually *nothing more* than its competitors. This may even be thought to be the most common case, otherwise attention would be arguably drawn on the extra value. While we have grown accustomed to this level of vagueness in advertising, encountering it in texts from which we expect actual information, such as an instruction manual, would result in strong awkwardness (Kaufer 1983).

#### Political discourse

**Fig. 7:**
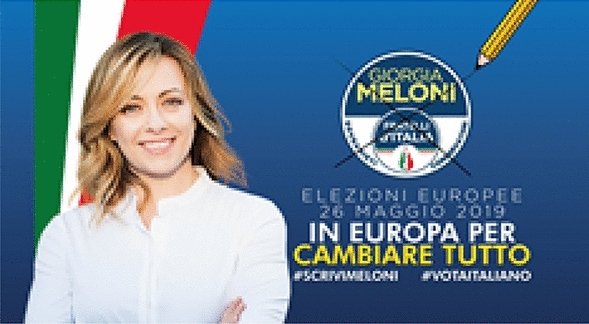
Meloni

**Fig. 8:**
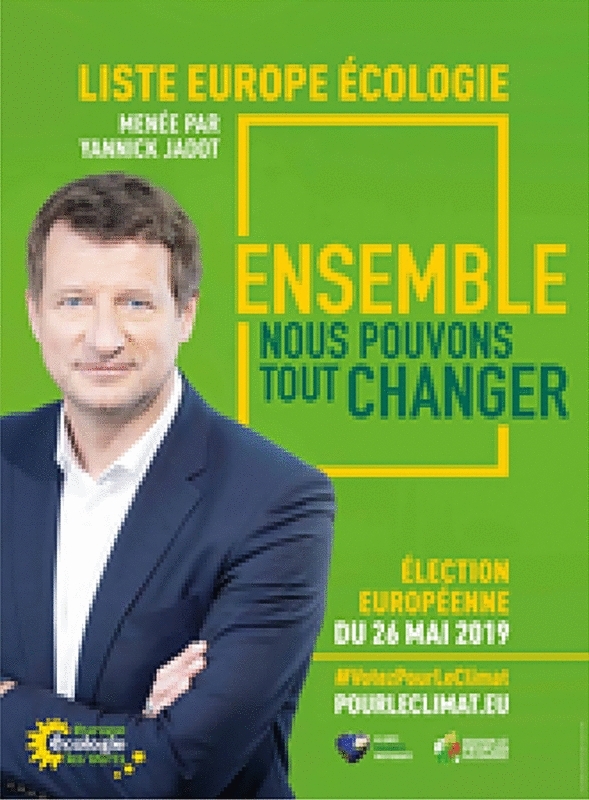
Jadot

Politicians make extensive use of vague expressions in their attempt to earn the people’s approval and votes. Here is an example of how vagueness is employed in exactly the same way by an Italian and a French politician (Giorgia Meloni from Fratelli d’Italia and Yannick Jadot from Europe Écologie Les Verts, respectively) during the 2019 European Parliament election campaign (Figs. 7, 8):

9. Giorgia Meloni: *In Europa per ****cambiare tutto*** (In Europe to **change everything).**
10. Yannick Jadot: *Ensemble nous pouvons ****tout changer* (**Together we can **change everything)**

In these posters, the reference of the vague (and hyperbolic) expressions “everything” is partially restricted by background knowledge of the parties’ different standpoints toward specific issues (such as migration policy, sustainability, safeguard of national identities, and so on). Still, the politicians do not actually commit to any specific future action. If they had been more specific, they probably would have garnered support from some, while disheartening others. Thanks to vagueness, they convey the positive connotation of a strong-willed figure who is ready to change what does not work within the European institutions, presumably catching the agreement of many people, even if they have strongly diverging opinions.

**Fig. 9:**
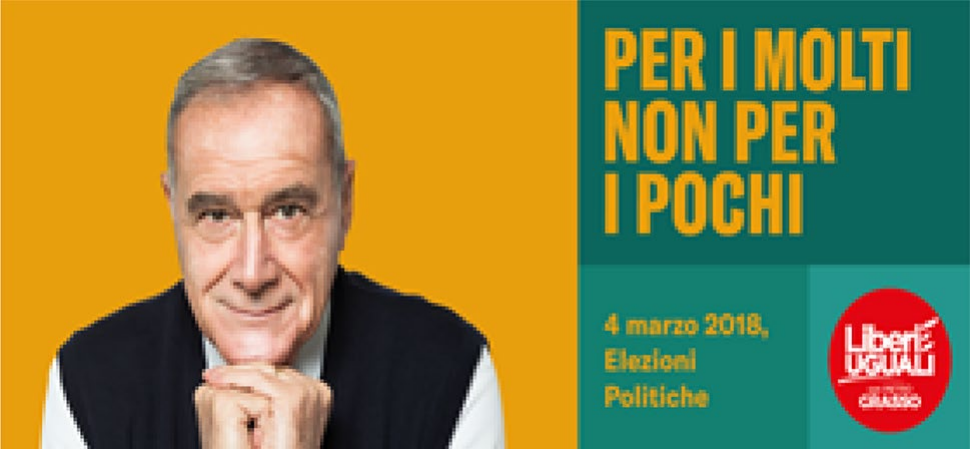
Grasso

In some cases, vagueness serves as a tool to criticize unspecified political opponents or their actions (Fig. 9).

11. Pietro Grasso: *Per ****i molti**** non per**** i pochi* (**For **the many** not** the few).**

In (11), Pietro Grasso, the founder of the Italian left-wing party Liberi e Uguali, states that his politics is not for *the few*, but for *the many*, probably drawing direct inspiration from a UK Labour party slogan. This raises the implicature that some politicians in Italy prioritize their own interests. Nevertheless, he does not specify who these people are (some secret lobbies? some politicians in particular? entrepreneurs? all of them, or only big ones?); and neither who *the many* are (all citizens or only the working-class people? only Italians or migrants too?), whose interests should be represented and protected. On the one hand, by using *the few* in a vague manner, the speaker decommits himself from attacking someone specifically, drastically reducing the likelihood of a counterattack; on the other hand, by employing the expression *the many*, without specifying particular groups, the speaker avoids the risk of making anyone feel excluded, potentially securing more support from voters. Once again, not all precisifications of these vague expressions are equally true or plausible in terms of whom the politician is actually committing to fight for.

One could argue that such generic expressions are required by the brief format of the advertisement and of the election post, where the punchline needs to be short, catchy, and memorable. Indeed, some authors have pointed out that implicit language (not only vagueness, but also presuppositions and implicatures) well responds to the constraint of conciseness imposed by these text genres (see Lombardi Vallauri 2019a). However, abundant instances of persuasive vagueness are also found in longer political speeches.^5^ Consider the following excerpt, taken from a speech held by François-Xavier Bellamy (representative of the French liberal-conservative party Les Républicains) running for the 2019 European Parliament elections:

12. (French): *Car qu'est-ce qui crée la crise environnementale? Nous ne la résoudrons pas si nous n’allons pas d’abord à la cause. ****Certains**** cherchent absolument à s'illustrer sur ce terrain alors**** ils**** en font le premier chapitre de**** leur programme et ils**** déclinent ****des solutions qui sont au fond pas si nouvelles*.**
  (English): For, what creates the environmental crisis? We are not going to solve it if we don’t get first at the cause. **Some** try by all means to show off on this field and **they** make it the first chapter of **their programme** and lay down **a list of solutions which are not so new after all.**

In (12), Bellamy’s criticism does not specify its recipients, utilizing the indefinite pronoun *certains* (*some*) and the anaphoric elements referring back to it. Furthermore, the omission of specific opponents allows him to remain vague about the nature of these inappropriate solutions and with respect to what these are allegedly “not so new” (with *new* being another positively connotated vague adjective largely used by politicians as opposed to the negatively connotated *old*, cfr. Lombardi Vallauri 2019a). Therefore, vagueness makes it possible for Bellamy, on the one hand, to (also) hint at some non-*bona fide* true contents, i.e., to implicitly accuse politicians who are not actually accountable of the described behavior; on the other hand, to generally frame his opponents in a negative aura, which in turn makes his plan about the environmental crisis more desirable. Here is a similar example from a speech held by Anna Baerbock (President of the German green party Bündnis 90/Die Grünen) during the same European Parliament campaign:

13. (German): *Aber, das müssen wir auch ganz klar sagen, es ist nicht alles gut in Europa, weil**** die Gegner der EU**** in den letzten Jahren auch ein zu leichtes Spiel hatten, weil ****die EU von einigen, eben wirklich nur als Freihandelszone betrachtet wird****, wo kein Platz ist für soziale Rechte und für einen sozialen Raum.*
  (English): But, we have to make it very clear, not everything is good in Europe, because **the opponents of the EU** have had too much of an easy time of it in recent years, because **the EU is really only viewed by some as a free trade area**, where there is no room for social rights and for a social space.

Similarly to Bellamy, Baerbock addresses her attacks to “some” generic “opponents of the EU,” whose behavior is also not further specified but still remains very negatively connotated. Again, the persuasive potential lies in the fact that although Baerbock does not take responsibility for that, recipients will also retrieve politicians who may not be guilty of disregarding social rights and social space.

Overall, the widespread presence of examples like the ones presented here suggests that marked vagueness seems to be a pervasive discourse strategy characterizing persuasive texts. Presumably, it provides speakers with several advantages. Indeed, by speaking vaguely, the speaker can avoid uttering and committing to appealing yet risky or non-*bona fide* true contents, promises or actions, still strategically conveying a clearly negative or positive connotation. We claim that the effectiveness of vagueness as a persuasion strategy arises from an interplay of cognitive and contextual factors, which will be discussed in the next section.

### The persuasive potential of vague expressions: diverting epistemic vigilance and shielding from responsibility

Various scholars have been investigating the persuasive potential of implicit linguistic strategies, relating them to an evolutionary advantage. Overtly trying to change someone’s view on something is usually perceived as intrusive and therefore triggers a critical attitude. Therefore, it is easier to convince someone of a content by conveying it implicitly. According to Reboul (2011), implicit communication may have specifically evolved to facilitate manipulation. In the case of vagueness, as the examples in "A pervasive strategy in persuasive texts" section have shown, referents are left implicit and for the addressees to reconstruct, which induces them to feel those contents as something they have arrived at themselves, and not something that originates externally (Mercier 2009:117–118). With regard to that, Lombardi Vallauri (2009, 2016) postulated a similarity between vagueness and implicatures, whose content also requires an inference on the part of the receiver, hence grouping them under the label “implicitness of content.”^6^ Therefore, whether the contents are retrieved by implicature or from vagueness, having the impression of being the author of certain conclusions generally makes people more prone to trust those contents without carefully scrutinizing them for accuracy. This tendency to trust more what comes from us is referred to as *egocentric bias*. People are generally “cognitive optimists” (Sperber et al. 1995:90), i.e., very confident about their own cognitive processes. Related to this, recent evidence suggests that people are significantly more likely to trust a speaker who implicates some false content rather than one that asserts or presupposes it (Mazzarella et al. 2018).^7^

This tendency to spare thorough checking may be due to the evolutionary preference for the least effort. “Fast and frugal” heuristics (Tversky and Kahneman 1974; Gigerenzer 2008) prove very helpful in emergency or dangerous situations and represent a reasonable compromise between rapid and accurate decision-making. Furthermore, as suggested by widely attested psycholinguistic phenomena such as semantic and pragmatic normalization and semantic illusions (Barton and Sanford 1993; Erickson and Mattson 1981; Ferreira 2003; Ferreira et al. 2002; Fillenbaum 1974; Frazier and Clifton 2015; Sanford 2002; Sanford and Graesser 2006; Sanford and Sturt 2002; Sturt et al. 2004), because of limited physiological and cognitive resources, linguistic inputs are generally processed in a somewhat shallow manner, constructing only *good-enough representations*. Christiansen and Chater (2016) suggest that the human brain must cope with what they call a *Now-or-Never-Bottleneck* in language processing: as new linguistic input rapidly comes in, there is not enough time nor cognitive resources to process every piece of information accurately, so some pieces of information must be elaborated quickly lest what comes next goes lost. As a consequence, it seems plausible that some contents receive a shallower examination than others (Lombardi Vallauri 2016).

Speakers can present linguistic information so as to influence the receivers’ choices of what to process more accurately and what in a shallower way. All these circumstances contribute to a fertile environment for vagueness to thrive as an implicit persuasive strategy. Indeed, as vague expressions leave people free to infer the implicit contents by themselves, those are likely to be processed in a shallower way, either (i) because recipients construct implicit contents themselves (as in the case of implicatures), thereby being less inclined to check their truth; or (ii) because they simply do not look for specific reference at all, process a vague expression mostly guided by its connotative apport, and focus their attention on other denotatively richer parts of the message. Our experimental application tries to explore if (ii) actually happens. Either way, vague expressions should presumably cause a decrease in the addressees’ *epistemic vigilance* (Sperber et al. 2010), that is to say, they should weaken the critical attention on some contents’ truth or trustworthiness, and in both cases decrease the chances for the implicit contents to be critically challenged. Chances that, we suggest, would be close to zero when no referent is retrieved, as the very generic denotative content alone is hardly likely to elicit a critical reaction.

This persuasive effect is made possible also by the fact that vagueness is a ubiquitous trait of our communication. As we are constantly confronted with underspecified expressions for us to interpret, this process generally goes unnoticed, even when the contents we are induced to construct are far from true. Applying these considerations to our society, it is fair to think that this process is even strengthened by present-day very fast information fruition, hindering critical and in-depth examination more than ever before.

Considering all this, an account of vagueness as a deliberate act of deresponsibilization on the part of the speaker (Caffi 1999, 2007, 2012, 2013) seems justifiable: following this approach, vagueness is one of the different ways of mitigating an act, which must be intended as a goal-oriented pragmatic strategy operating on the propositional level aimed at reducing (i) one’s enunciative obligations and (ii) one’s interactional risks.

As this brief literature review has shown, vagueness arguably derives its persuasive potential from an interplay of cognitive mechanisms and contextual factors, resting both on the exploitation of the egocentric bias and the elusion of precise contents that could be recognized as false. In order to confirm these intuitions, and in particular to test to what extent recipients of vague expressions attempt to retrieve more precise referents that possibly remained implicit, we carried out an online experiment involving reading times measurement, which will be described in Sect. "Experimental application."

## Experimental application

### Background and Research Hypothesis

The considerations presented in the previous section about vague expressions’ persuasiveness need to be substantiated by empirical inquiry. Indeed, linguistic vagueness is a phenomenon that has received significant theoretical discussion over the last decades, and yet such discussion has been supported by poor experimental checking. Most experiments conducted to explore the interpretation and processing of vague expressions’ are of the behavioral, offline type (Alxatib and Pelletier 2011; Bonini et al. 1999; Égré and Zehr 2018; Serchuk et al. 2011; Solt 2018). The most employed paradigms are probably decision-making and judgment elicitation tasks addressing vague predicates. As an example, in several tests participants were asked questions like: “When is it true that a man is *tall*?”, “When is a man *old?”* (italics ours), (Bonini et al. 1999; Serchuk et al. 2008, 2011; Alxatib and Pelletier 2011; Égré and Zehr 2018). However, these paradigms do not shed light on the real-time processing of vague expressions. This analysis is essential for understanding whether and to what extent addressees in online language processing retrieve and critically evaluate the various potential meanings conveyed by vague expressions.

**Fig. 10:**
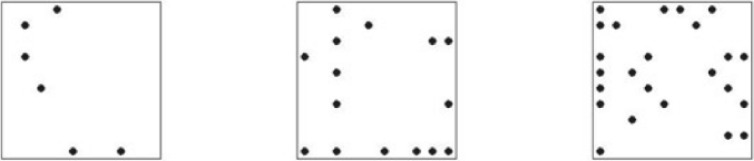
Stimuli sample from Green and van Deemter (2019:71)

In this context, an exception is Green and van Deemter’s (2019) experimental study, which employed a response-elicitation task with vague expressions. The authors tested the so-called cost reduction hypothesis, i.e., «the idea that vague expressions are easier to process, by a speaker and/or a hearer, than expressions that are not vague (i.e., crisp)» (ivi:64). In their experiments, they would present pictures like the following ones (Fig. 10):

Participants were asked either «Choose the square with 6 dots» or «Choose the square with about 10 dots». Their experiments reported that vague instructions resulted in faster responses compared to crisp instructions. However, the authors did not take this result as confirming the cost reduction hypothesis, as the response time difference was not consistent across all tasks, and because they deem that other factors may have played a role, as, for example, the subject’s expertise in the tested competence (e.g., familiarity with precise numbers). By the way, a common factor in the studies presented above is that vague expressions have been mostly tested in isolation, and the role of the context has generally not been taken into account.

The experiment we present here specifically sets out to start exploring processing times of vague expressions, in order to see whether they receive different attention and/or require specific cognitive effort on the part of receivers, as compared to more precise expressions.

To this end, we tested reading times associated, respectively, with vague vs. precise expressions in *precising* vs. *non-precising* contexts ("Method" section) See an example of a vague target preceded by the two levels of context precisation in (16) and (17):

### Precising context

(16) (Italian, original) Nell’aula in cui si tiene la lezione di diritto privato ci sono *cinquanta* studenti e *quaranta* sedie. Il professore chiede agli alunni dell’ultima fila di andare a prendere delle sedie nell’aula a fianco.

(English) In the classroom in which the private law course takes place, there are *fifty* students and *forty* chairs. The professor asks the students sitting in the last row to go and take some chairs in the adjacent classroom.

### Non-precising context

(17) (Italian, original) Nell’aula in cui si tiene la lezione di diritto privato ci sono *molti* studenti e *poche* sedie. Il professore chiede agli alunni dell’ultima fila di andare a prendere delle sedie nell’aula a fianco.

(English) In the classroom in which the private law course takes place, there are *many* students and *few* chairs. The professor asks the students sitting in the last row to go and take some chairs in the adjacent classroom.

Varying the level of context precisation enabled us to explore reading behaviors associated with two different levels of vagueness, namely, vague expressions enriched by precising context vs. vague expressions not enriched by context—an aspect that, to the best of our knowledge, has not received much experimental attention yet. Our experimental application, therefore, explores the interplay between target underspecification and context informativeness, and our research questions are the following:

1. Are reading times (henceforth, RTs) of vague expressions preceded by a non-precising context significantly different from RTs of the same vague expressions preceded by a precising context? And, similarly, are RTs of vague expressions preceded by a precising context significantly different from RTs of precise expressions in the same contexts?
2. If yes, how are these differences oriented? Is there an increase of RTs along with the level of vagueness, indicating that addressees devote additional effort to vague expressions (possibly in order to retrieve at least some of the specific meanings they potentially and implicitly include?). Or do RTs increase with an increase of informativity, indicating major interpretation effort to process precise information, and lesser effort with vagueness (consistent with the cost reduction hypothesis)?

These questions aim to investigate two primary aspects related to vague expressions elaboration: (i) whether different levels of context precisation display a cognitive characterization in terms of processing effort (Question 1); and (ii) whether vague expressions require additional cognitive effort, possibly due to an integration of the implicit content on the part of addressees), or if, instead, they elicit a more economic type of processing (suggesting that referents left implicit are not sought for). In case the results suggest a positive answer to the first question (significant difference among different levels of vagueness), the individuation of different levels of vagueness would find some psycholinguistic support. Assuming the first hypothesis to be confirmed, as for the second question we suggest that, if longer RTs were to be associated with vaguer stimuli, we may conclude that when vague expressions are read, recipients generate an inference to replace the denotatively poor expression with some (more precise) semantic meaning; if, conversely, the trends display an increase of RTs along with the level of precision, this would suggest that a minor effort is devoted to look for the possible referents of the vague expression. The latter outcome would provide cognitive support to our argument for considering vagueness a persuasive implicit strategy, as we suggest that without constructing and processing a precise meaning, the likelihood of a critical reaction is very low. In the following section, we will present the experimental design, followed by a formalization of our hypotheses.

### Method

#### Materials and Hypotheses

We created 33 short texts, each one consisting of one or two context sentences followed by a target sentence. Context sentences were either precising or non-precising, and target sentences contained either vague or precise expressions.

We decided to manipulate only one parameter at a time in each condition, and therefore, the two parameters (precising vs non-precising context; vague vs. precise target) were combined into three experimental conditions (Table 1):

A. Non-precising context—vague target
B. Precising context—vague target
C. Precising context—precise target

**Table 1:** Experimental conditions

Context	Target
Non-precising	Vague
Precising	Vague
Precising	Precise

Future research could consider incorporating a fourth experimental condition, presenting non-precising context and precise target (let us say, condition D). Considering the exploratory nature of our study and the necessity to maintain a manageable experiment duration, this condition was omitted from the experimental design. The experiment is primarily intended to assess the impact of context on the processing of vague expressions, rather than precise ones. Precise expressions represent self-sufficient semantic units that can be interpreted without having recourse to contextual information and are therefore likely to be processed essentially in the same way, regardless of the context being precising or non-precising.^8^

Hence, a total of 99 short texts were generated. We then divided the short texts into three different lists (i.e., 1, 2, and 3) in counterbalancing them by condition (Table 2), so that each participant was presented with 11 short texts per condition, and never with the same text in different conditions. The presentation order was entirely randomized for each participant.

**Table 2:** Stimuli (examples)

**Non-precising** context—**vague** target	**Precising** context—**vague** target	**Precising** context—**precise** target
Francesco deve allestire la tavola per la festa di compleanno del fratello. Ci saranno **molti** ospiti e sul tavolo ci sono **pochi** bicchieri. Francesco va in cucina e prende **dei** bicchieri	Francesco deve allestire la tavola per la festa di compleanno del fratello. Ci saranno **dieci** persone e sul tavolo ci sono **otto** bicchieri. Francesco va in cucina e prende **dei** bicchieri	Francesco deve allestire la tavola per la festa di compleanno del fratello. Ci saranno **dieci** persone e sul tavolo ci sono **otto** bicchieri. Francesco va in cucina e prende **due** bicchieri
Francesco is setting the table for his brother’s birthday party. There will be **many** guests and on the table there are **few** glasses. Francesco goes in the kitchen and takes **some** glasses	Francesco is setting the table for his brother’s birthday party. There will be **ten** guests and on the table there are **eight** glasses. Francesco goes in the kitchen and takes **some** glasses	Francesco is setting the table for his brother’s birthday party. There will be **ten** guests and on the table there are **eight** glasses. Francesco goes in the kitchen and takes **two** glasses
Negli ultimi mesi Giulio è stato **un pessimo studente.** I suoi genitori stanno cercando di capire quale possa essere la causa, ma sono molto preoccupati e non sanno cosa fare. Temono **conseguenze serie**	Negli ultimi mesi Giulio ha avuto **l’insufficienza in tutte le materie**. I suoi genitori stanno cercando di capire quale possa essere la causa, ma sono molto preoccupati e non sanno cosa fare. Temono **conseguenze serie**	Negli ultimi mesi Giulio ha avuto **l’insufficienza in tutte le materie**. I suoi genitori stanno cercando di capire quale possa essere la causa, ma sono molto preoccupati e non sanno cosa fare. Temono **una bocciatura**
Over the last months Giulio has **been a terrible pupil**. His parents are trying to figure out what can be the reason for it, they are very worried and do not know what to do. They fear **serious consequences**	Over the last months Giulio has **failed most subjects**. His parents are trying to figure out what can be the reason for it, they are very worried and do not know what to do. They fear **serious consequences**	Over the last months Giulio has **failed most subjects**. His parents are trying to figure out what can be the reason for it, they are very worried and do not know what to do. They fear **a school failure**

For standardization purposes, all texts/stimuli were built by keeping their length between 27 and 33 words. In greater detail, the difference in the number of words of the three lists was checked through a non-parametric Friedman test of differences that failed to reach statistical significance (*χ*^*2*^ = 0.16, *p* = 0.92).

As concerns the vagueness level, the Non-precising context—vague target and the Precising context—vague target conditions counted 947 words in total each (Median = 30 in both cases), whereas the Precising context—precise target condition counted 932 words (Median = 30). The same non-parametric Friedman test of differences revealed that the differences among the three conditions were not significant (*χ*^*2*^ = 2.23, *p* = 0.33). See an example of two stimuli in the following picture (originals in Italian):

As can be seen, the stimuli were characterized by out-of-the-blue contexts, which were controlled to match the subjects’ assumed shared knowledge (Bochnak and Matthewson, 2020). Therefore, the majority of texts had a fictional character. Proper nouns referring to actual referents were chosen among those plausibly known by all participants (e.g., *Leonardo Da Vinci, Carlo Magno, Venezia*). All the lexical items used are part of the Italian basic vocabulary for both vague and precise targets, thus eliminating the potential disturbance of frequency effects.

For the creation of precise vs. vague targets, we attempted to associate two maximally semantically close expressions; i.e., we paired each precise expression with the corresponding vague equivalent that closely matched its representation. The most frequently used patterns are as follows: where the precise expression is a precise numeral, the vague variant is an indefinite (e.g., two → some); when the more precise is a hyponym or a subordinate-level category, the vaguer is its hypernym or an immediate superordinate-level category (e.g., appendicitis → inflammation; failing most subjects → being a terrible pupil); when the more precise is a common noun (e.g., a cake) the vaguer equivalent is an indefinite noun (e.g., something). The full list of stimuli is available in Appendix.

Given the above-presented conditions, we hypothesized that the reading times (RTs) of conditions A, B, and C are significantly different from each other (i.e., RTs A ≠ RTs B ≠ RTs C). Specifically, should this first hypothesis be verified, we hypothesize that the RTs of the three conditions display an increase from A to B to C (i.e., RTs A < RTsB < RTsC).

#### Natural reading

In order to deal with the possibility that the reading activity of participants was affected by a habituation effect, and that the participants tend to skip the reading to finish the experiment in a shorter amount of time, three versions of the experiment were developed. In the first version (i.e., maximal reading naturalness and minimal control), the task simply consisted in reading each short text and pressing a button after having read it. In the second version (i.e., average naturalness and average control), 6 out of the 33 randomly presented short texts were followed by a true/false comprehension mandatory question (albeit not specifically about the vague/precise target, as the target varied across the three conditions) In doing so, the participants did not know in advance which sentence could be followed by a question. Finally, in the third version (i.e., minimal naturalness and maximal control), all the short texts were followed by a simple question, thus requiring maximal attention throughout the task. The presence of an explicit task is a relevant parameter when testing language processing. As already mentioned, Fernanda Ferreira and colleagues’ theory of “good-enough” representations (supported by significant evidence) suggests that language processing is often based on shallow heuristics, and often yields merely “good-enough,” i.e., partial and incomplete semantic representations. Specifically, Karimi and Ferreira explain: «representations formed during language processing are sometimes just “good enough” *for the task at hand*» (Karimi and Ferreira 2016:1013, emphasis added). Thus, we could assume that the more explicit and demanding the task, the greater attention would be paid to text elaboration. Hence, we tested whether and to what extent the presence of explicit and diversely demanding tasks impacted vague expressions processing.

In other words, we devised inserting questions for two main purposes: on the first level, to prevent shallow, inattentive, or unreliable reading behaviors. Secondly, on a more operative level, the questions were added to assess each participant’s performance and subsequently exclude those who had poor correct answers rates, which suggests a general lack of attention while taking the experiment.

**Fig. 11:**
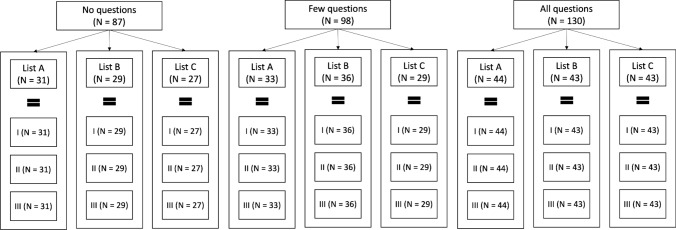
Experimental design. *Note*. I, II, and III represent the vagueness levels within each list. Each level contains 11 short texts. The lists, indicated with the letters A, B, C, are identical in all experiments

To sum up, the complete experimental design comprised three different experiments (thus, three different samples) differing in the number of checking questions provided (i.e., 0, 6, 33). In each experiment, the participants were presented with the same three lists of short texts in a randomized order (Fig. 11).

#### Procedure

Due to the Covid-19 pandemic situation, we built an online procedure. By accessing a single non-reusable link,^9^ participants could run the experiment from home on their laptops, smartphones, or tablets. Participants have been recruited by means of Roma Tre University mailing lists. The participation was on a voluntary basis and no reward or credit was provided.

An introductory screen presented a brief description of the task and the informed consent. Later on, participants were presented with a training phase of 5 short texts comparable by length, structure, and content to those in analysis. Subsequently, the true test started. To favor natural reading, the texts (13-point Arial font) were presented to the participants in a fully randomized order and with no interruptions among them, except for the questions (in Experiment 2 and 3). Participants were instructed to read at their own pace and to press a blue arrow at the bottom of the page as soon as they had finished reading. Right after that, the next text (or question) appeared. The arrow was placed in a fixed position throughout the texts and questions. The reading time count started from page load and ended when the participant clicked on the arrow. In the experiments where questions appeared, the participants had to answer by clicking on the correct response and confirm their answer by clicking on the same arrow. This procedure ensured that the mouse was already on the arrow and prepared to click once again at the end of the following text.

**Fig. 12:**
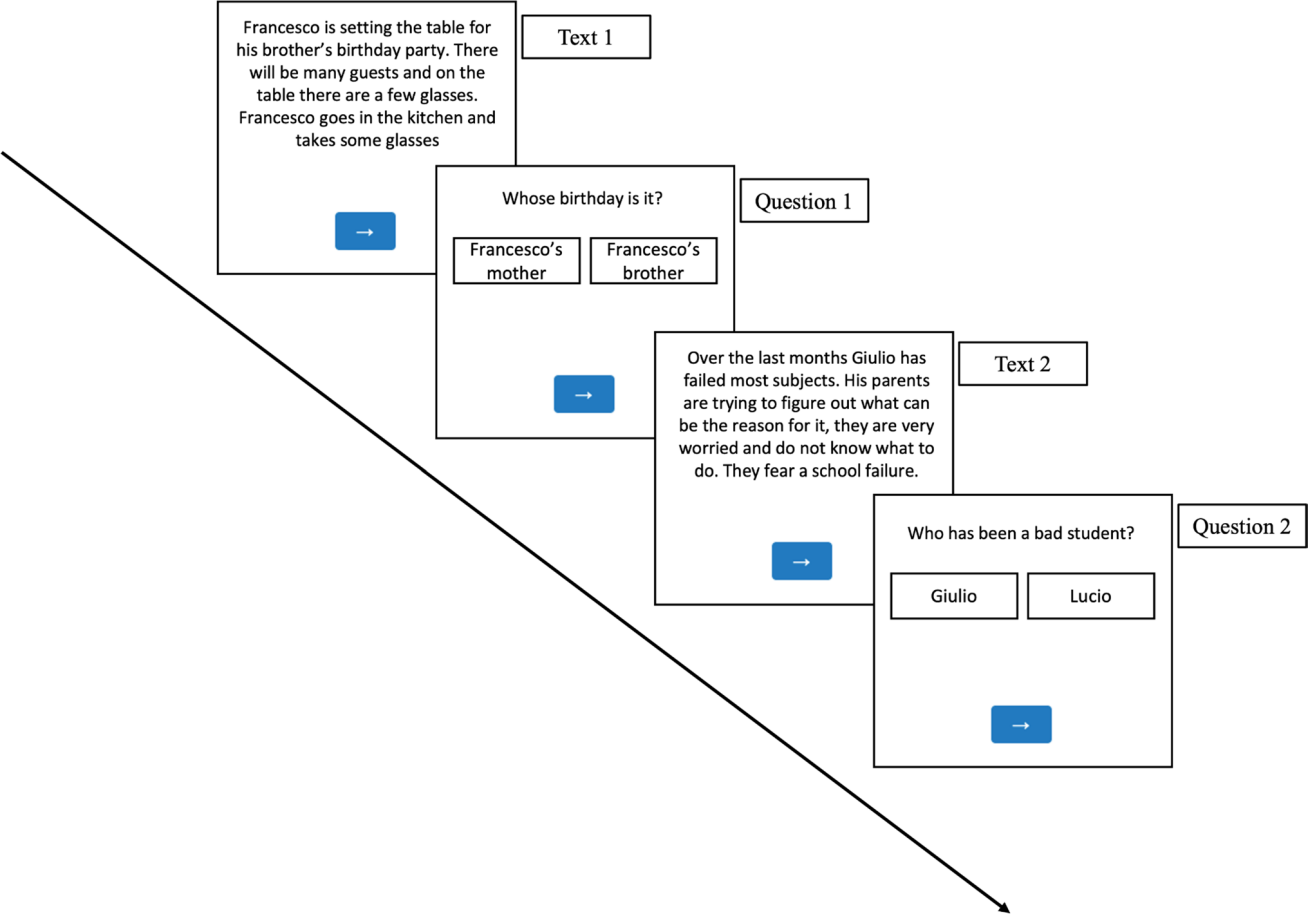
Experimental procedure. *Note*. The questions were only present in Experiments 2 and 3

Participants who accessed the link via smartphones and tablets did not have to use a mouse; they simply clicked on the same fixedly placed arrow to proceed. Figure 12 displays a representation of the procedure.

#### Participants, exclusion criteria, and preliminary data analysis

As a starting point, we recruited 392 Italian mother tongue participants. Later on, given the lack of control intrinsic to any online experiment, we applied several exclusions according to the following pre-established criteria:

- The percentage of correct answers had to be > 50%; thus, 30 participants were discarded due to this criterion.
- In order not to take the results from participants whose attention may have been distracted by something having nothing to do with the experiment, all participants who had completed the whole task in more than 15 min (roughly speaking twice the median) were excluded from the analysis. This criterion was chosen in accordance with Revilla and Höhne (2020) recommendations on the ideal length of an online survey (i.e., between 10 and 20 min). We excluded 45 more participants because of this criterion.

After such exclusions, our sample counted 315 participants (*M*_age_: 34.00 *SD* = 13.00, females 61%). The average duration of the experiment completion was 8.05 min (*SD* = 2.50, *Mdn* = 7.86 min). Not surprisingly, the average duration significantly increased with the number of presented questions (Table 3), *p* < 0.001.

**Table 3:** Distribution of the participants and experiment duration

	List					
Exp	1	2	3	Total	Duration (Mean)	Duration (Median)
No questions	31	29	27	87	379 s	369 s
Few questions	33	36	29	98	495 s	481 s
All Questions	44	43	43	130	545 s	541 s
Overall	108	108	99	315	483 s	472 s

The participants were divided as in Table 3; the distribution has proved to be balanced, χ^2^ = 0.53, *df* = 4, *p* = 0.97.

### Results

For the statistical analyses, IBM SPSS 26.0 (IBM Corp) and Jamovi (The jamovi project, 2022) were used. In particular, the generalized linear mixed effect model was implemented through the GAMLj module (Gallucci 2022). The significance level was set to α = 0.05. The plot was made by means of R’s Flexplot package (Fife 2022). For significant results, the 95% confidence intervals are provided.

Several steps have characterized the data analysis. In the following, we summarize each passage:

As a first step, we performed data trimming on the RTs (Berger and Kiefer 2021). As for the lower threshold, we excluded all trials whose duration was < 2000 ms. For the upper threshold, the trials whose duration was higher than the mean + 2 SD (Zimmerman and Williams 2000) were trimmed.

At this point, an assessment of the overall data quality proved that it was fairly high: the mean percentage of valid trials of the sample was 90.8% (*Mdn* = 96.9% *SD* = 17.6%). In greater detail, 90% of the participants had more than 80% valid trials. However, to further increase the quality, we removed from the analyses all the participants whose percentage of valid trials was less than 50% (*N* = 13). After such exclusions, the average percentage of valid trials increased to 94.2% (*Mdn* = 96.9% *SD* = 8.6%). Specifically, 93.3% of the participants had more than 80% valid trials, whereas 80.4% had more than 90% valid trials.

Subsequently, to minimize the individual differences in the reading times RTs possibly due to the different devices employed by the participants (and the difference between fast-readers and slow-readers), the RTs were parameterized in a subtractive fashion (Marini et al. 2022), namely by subtracting, for each trial, the whole task average RT of that participant to the RT of the single trial, as follows:

$$Parameterized\,RT\left(trial X\right)=RT\left(trialX\right)-Participant\,Avg. RT$$

Such a procedure allowed us to have, for each trial, the extent to which it required more or less time to be read with respect to the average reading time of that participant.

Aiming at statistical parsimony, we condensed all the analyses into a single model that took into consideration our manipulations, namely:

- **Vagueness level**: non-precising context—vague target (i.e., condition A), precising context—vague target (i.e., condition B), and precising context—precise target (i.e., condition C).
- **Experiment**:
  - *no questions*: maximal naturalness and minimal control
  - *few questions*: average naturalness and average control
  - *all questions*: minimal naturalness and maximal control.

Given the non-normality of the RTs, a Generalized Linear Mixed Effect Model (GLMM) with random subject and stimulus intercepts was planned, having the parameterized RT as the model-dependent variable (i.e., target variable). In accordance with Lo and Andrews (2015), Gamma distribution was used to “statistically mimic RT responses,”^10^ and the inverse link function was chosen because of the superior fit with RTs reported by Balota et al. (2013) in a psycholinguistic context. The model assessed the fixed effects of three factors and one covariate. The three effects were vagueness level, experiment, and their interaction. As for the covariate, we added to the model the difference in the number of words across the three vagueness levels, taking the precising context—precise target condition as the baseline.

Through this procedure, we aimed to find an effect of the vagueness level and, at the same time, make sure that the differences among the experimental tasks (i.e., number of questions) and the texts’ different length had not affected the RTs.

As hypothesized, the effect of the experimental procedure was not significant, *χ*^*2*^ = 1.66, *p* = 0.436. Similarly, the interaction effect of the experiment and the vagueness was not significant, *χ*^*2*^ = 7.20, *p* = 0.125. The significant effects were those of the vagueness level, *χ*^*2*^ = 17.60, *p* < 0.001^11^ and the difference in words number, *χ*^*2*^ = 6.97, *p* = 0.008 (Table 4).

**Table 4:** Significant Fixed effects parameter estimates

Names	Effect	Estimate	*SE*	95% confidence interval		z	*p*
				Lower	Upper		
(Intercept)	(Intercept)	1.994	0.042	1.911	2.077	47.299	< .0001
Condition 1	A—B	0.018	0.008	0.003	0.033	2.293	0.0219
Condition 2	B—C	0.012	0.008	-0.003	0.027	1.599	0.1099
WDiff	WDiff	-0.012	0.005	-0.021	-0.003	-2.640	0.0083

The contrasts of the condition factor are “sequential forward differences” (i.e., adjacent levels are compared: A vs B and B vs C)

**Fig. 13:**
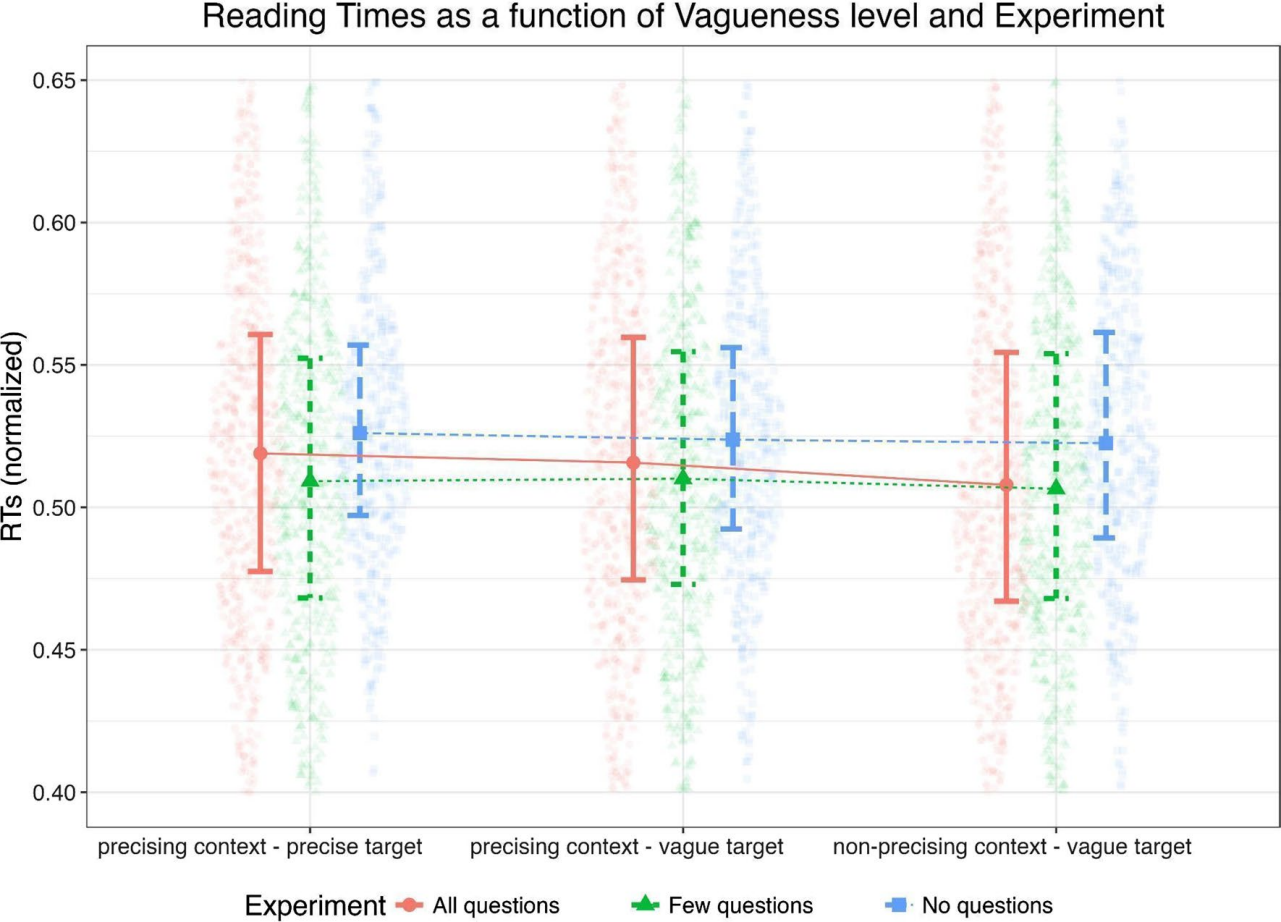
Reading times as a function of vagueness level and experiment. *Note*. The form of the violins indicates the distribution curve. Whiskers are drawn from the first quartile to the third quartile

As compared with the non-precising context—vague target condition (*M* = 0.498, 95% CI [0.478,0.519], *SE* = 0.010), the precising context—vague target (*M* = 0.502, 95% CI [0.482,0.524], *SE* = 0.010), and the precising context—precise target (*M* = 0.505, 95% CI [0.485,0.527], *SE* = 0.010) conditions led to an increase of the RT. The mean values can be seen in Table 5 and Fig. 13.

**Table 5:** Mean reading times as a function of the vagueness level (Estimated Marginal Means)

Condition	Mean	*SE*	95% confidence interval	
			Lower CI	Upper CI
non-precising context—vague target	0.498	0.010	0.478	0.519
precising context—vague target	0.502	0.010	0.482	0.524
precising context—precise target	0.505	0.010	0.485	0.527

More in detail, the a priori repeated contrasts highlighted that the difference between the non-precising context—vague target and the precising context—vague target conditions approached significance (*p* = 0.109), as opposed to the remaining differences, which reached it. When correcting through Holm–Bonferroni correction, the significance pattern remained unvaried (Table 6).

**Table 6:** Pairwise comparisons

Post hoc comparisons—vagueness level			Difference	*SE*	Z	*p*_contrasts_	*p*_holm_
Comparison							
Condition		Condition					
Precising context—vague target	–	Precising context—precise target	0.012	0.007	1.60	0.109	0.109
Non-precising context—vague target	–	Precising context—vague target	0.017	0.007	2.29	0.021	0.043
Non-precising context—vague target	–	Precising context—precise target	0.029	0.007	4.02	< .001	< .001

Furthermore, an examination was conducted to evaluate the potential influence of the diverse devices utilized in the experimental setup. Specifically, an alternative model was constructed, incorporating the devices as a random effect. Notably, the inclusion of the device random effect did not introduce any discernible variance (*SD* = 0.002), while the fixed effects remained unchanged.

## Discussion

Our data reported an increase in RTs along with the informativity level, RTs of condition A being below the overall average. RTs means of condition A were significantly different from the other two conditions (while no significant difference was reported between RTs of conditions B and C). This trend, consistent with the cost reduction hypothesis, seems to confirm that vague expressions undergo a more economic and shallow type of processing, during which receivers probably do not devote specific effort to look for precise referents that the source may have alluded to.

More generally, our results seem to suggest that utterance interpretation is governed by a presumption of lexical-pragmatic acceptability and that anomaly detection works in a top-down way, its threshold being semantic inconsistency, and not semantic underdetermination or incompleteness per se (as shown by the lower-than-average RTs of condition A), thereby confirming that underdetermination is cognitively treated as a default condition of communication (Gibson et al. 2019; Piantadosi et al. 2012; Scott-Phillips 2014; Sperber and Wilson 2001; Voghera 2012). Our findings, indeed, also support accounts of vagueness which suggest that this feature represents an economic communicative strategy. Abundant evidence has come to show that communication generally occurs by means of “good-enough” meaning representation and “shallow processing” (Barton and Sanford 1993; Ferreira et al. 2002; Ferreira 2003; Sanford and Sturt 2002; Sturt et al. 2004; Sanford 2002; Sanford and Graesser 2006). Our results seem to align with these theories, *a fortiori* given our results’ cross-experimental consistency. Despite the presence of different tasks, requiring different levels of attention on the part of participants, indeed, all experiments displayed the same reading behavior trends, with vagueness being consistently an economic factor. The use of different devices proved to be nonsignificant as well (consistently with Vyshedskiy et al. 2022; a.o.).

As a consequence, crucially for the present work, this interpretation of the data appears to support the hypothesis that vagueness serves as an effective means of persuasion by potentially diverting attention from questionable content details. Results are therefore in line with phenomenological claims made on the topic (Lombardi Vallauri 2016, 2019a). In psycholinguistic research, it is widely acknowledged that linguistic material which is processed faster is allotted minor attention and energy (Hornby 1974; Loftus 1975; Schwarz 2016). As explained in "The persuasive potential of vague expressions: diverting epistemic vigilance and shielding from responsibility" section, prior research also suggests that a reduction in cognitive load comes along with a decrease in epistemic vigilance, i.e., the tendency and effort to detect fallacies and implausibility, a decrease which makes critical challenge or rejection of contents less likely.

Our results suggest that since vague expressions trigger shorter RTs as opposed to their precise counterparts, they are probably processed in a shallower way, i.e., without or feebly seeking more precise referents. As a consequence, critical reaction is less likely to arise toward questionable contents that are potentially included in the vague reference, but at most approximately and superficially retrieved by addressees. In conjunction with the widespread use of vague expressions in persuasive texts (as shown in "A pervasive strategy in persuasive texts" section), this seems to support the idea that vagueness can make it easier for persuaders (such as politicians and advertisers) to convey highly connotative yet poorly denotative questionable information, thereby avoiding critical reaction or rebuttal. This means that the wide exploitation of vagueness in public communication enhances the approval of messages potentially including shallow representations of questionable, exaggerated, or false information, with possible long-term consequences (in terms of votes or sales figures).

We are aware that our study presents some limitations, and even though most were due to the need to serve experimental purposes, some adjustments in future research can still be envisaged. Methodologically, a higher level of accuracy will likely be reached by means of eye-tracking experimental methodology, which, as already mentioned in "Method" section, at the moment of inquiry was infeasible due to the sanitary situation resulting from the Covid-19 pandemic. Furthermore, in future works it may be useful to also have data about the fourth condition (namely: non-precising context—precise target), which was not considered for the present work, given our interest in exploring the different elaboration of vague targets in differently precising contexts.

As far as materials are concerned, more balanced stimuli may be desirable: for this purpose, future research may involve a qualitative balancing of semantic vagueness triggers (e.g., indefinite pronouns, general extenders, etc.), and possibly also the inclusion of syntactic vagueness stimuli, in order to identify any possible effect of specific triggers on subjects’ RTs. Obviously, the same experiment could be made with languages other than Italian, thereby inquiring about possible cross-linguistic differences in the reading behaviors of vague expressions, which could be ascribed to typological language features (e.g., morphosyntactic marking of indefiniteness) and/or, in turn, to cultural tendencies. This might improve the analysis of specific discursive practices and foster cross-cultural research, especially with regard to persuasive language.

In summary, due to the explorative nature of the present experimental work and its inherent limitations, further research is needed to explore these phenomena in both persuasive and non-persuasive contexts.

## Conclusion

This work represents a contribution to the study of linguistic vagueness, notably focusing on its working as a persuasive implicit strategy. We suggested that high levels of vagueness can serve as an implicit linguistic strategy. By leaving content unspecified, vague expressions allow recipients to construct the meaning they prefer, ensuring shallow processing and thereby reducing the risk of critical evaluation. In making these assumptions, we were supported by relevant cognitivist literature and by the high frequency of vague expressions in predominantly persuasive texts (advertisements and political slogans and speeches) in different languages. To experimentally test the cognitive behavior underlying the processing of more or less vague expressions, we carried out a threefold study in which we measured reading times (RTs) of vague expressions in precising vs. non-precising contexts in short texts in Italian. Results showed: (i) a significant difference among the two levels of vagueness, providing support for our characterization; consistently, (ii) an increase in RTs along with the level of precision, which indicates that vaguer expressions received more economic processing (as suggested by the cost reduction hypothesis), presumably because a minor effort was devoted to retrieving precise referents, thereby suggesting a shallow type of elaboration.

Taken together, our results provide support to the cognitivist hypothesis that vagueness represents an effective implicit means of persuasion, and are therefore in line with previous research work: due to a decrease in epistemic vigilance, a possible distraction from questionable content details seems to be more likely to occur when a vague expression is used instead of a precise one.

Our study encourages further both theoretical and experimental investigations of this kind. The findings from such inquiries can effectively guide us in interpreting and detecting manipulative uses of implicit communication in the public domain.

## Data Availability

The stimuli and data that support the findings of this study will be publicly available on osf.io upon acceptance.

## Appendix

**Table:**

Condition		
Non-precising context—vague target	Precising context—vague target	Precising context—precise target
Leo deve allestire la tavola per la festa di compleanno del fratello. Ci saranno molti ospiti e sul tavolo ci sono pochi bicchieri. Leo va in cucina e prende** dei bicchieri**	Leo deve allestire la tavola per la festa di compleanno del fratello. Ci saranno dieci persone e sul tavolo ci sono otto bicchieri. Leo va in cucina e prende** dei bicchieri**	Leo deve allestire la tavola per la festa di compleanno del fratello. Ci saranno dieci persone e sul tavolo ci sono otto bicchieri. Leo va in cucina e prende** due bicchieri**
Le biografie di Carlo Magno raccontano che egli ebbe molti figli. Secondo alcuni racconti, **più della metà di loro** morirono prima di lui a causa delle scarse condizioni igieniche	Le biografie di Carlo Magno raccontano che egli ebbe diciotto figli. Secondo alcuni racconti, **più della metà di loro** morirono prima di lui a causa delle scarse condizioni igieniche	Le biografie di Carlo Magno raccontano che egli ebbe diciotto figli. Secondo alcuni racconti, **dieci di loro** morirono prima di lui a causa delle scarse condizioni igieniche
Nell’aula in cui si tiene la lezione di diritto privato ci sono molti studenti e poche sedie. Il professore chiede agli alunni dell’ultima fila di andare a prendere **delle sedie** nell’aula a fianco	Nell’aula in cui si tiene la lezione di diritto privato ci sono cinquanta studenti e quaranta sedie. Il professore chiede agli alunni dell’ultima fila di andare a prendere **delle sedie** nell’aula a fianco	Nell’aula in cui si tiene la lezione di diritto privato ci sono cinquanta studenti e quaranta sedie. Il professore chiede agli alunni dell’ultima fila di andare a prendere **dieci sedie** nell’aula a fianco
Due ragazzi non ancora maggiorenni cercano di comprare delle sigarette in un tabaccaio. Il venditore verifica la loro età e gli dice di tornare **tra un po’ di tempo**	Due ragazzi di diciassette anni cercano di comprare delle sigarette in un tabaccaio. Il venditore verifica la loro età e gli dice di tornare **tra un po’ di tempo**	Due ragazzi di diciassette anni cercano di comprare delle sigarette in un tabaccaio. Il venditore verifica la loro età e gli dice di tornare **tra un anno**
Un tuo amico ha deciso di organizzare una sorpresa per la sua fidanzata. Sa di non essere un tipo molto creativo e ti chiede di dargli una mano a preparare** delle cose**	Un tuo amico ha deciso di organizzare una festa a sorpresa per la sua fidanzata. Sa di non essere un tipo molto creativo e ti chiede di dargli una mano a preparare** delle cose**	Un tuo amico ha deciso di organizzare una festa a sorpresa per la sua fidanzata. Sa di non essere un tipo molto creativo e ti chiede di dargli una mano a preparare** la sala**
Leo sta facendo un corso di cucina e dopo una lezione vuole cucinare qualcosa per esercitarsi. Prima di iniziare, dispone **gli strumenti** sul tavolo e spegne il telefono per non essere disturbato	Leo sta facendo un corso di cucina e dopo una lezione vuole cucinare una torta per esercitarsi. Prima di iniziare, dispone **gli strumenti** sul tavolo e spegne il telefono per non essere disturbato	Leo sta facendo un corso di cucina e dopo una lezione vuole cucinare una torta per esercitarsi. Prima di iniziare, dispone **le ciotole e la frusta** sul tavolo e spegne il telefono per non essere disturbato
Circa il venticinque percento degli italiani presenta intolleranze alimentari. Quando persone con intolleranze vanno in un ristorante sperano di trovare pietanze **adatte alla loro salute**, ma non sempre ci riescono	Circa il venti percento degli italiani presenta un’intolleranza al lattosio. Quando persone con questa intolleranza vanno in un ristorante sperano di trovare pietanze **adatte alla loro salute**, ma non sempre ci riescono	Circa il venti percento degli italiani presenta un’intolleranza al lattosio. Quando persone con questa intolleranza vanno in un ristorante sperano di trovare pietanze **senza lattosio**, ma non sempre ci riescono
Il Paese versa in una grave crisi economica. La prima operazione del nuovo governo sarà un intervento nel sociale. Molti cittadini pensano che sia una decisione giusta, perché aiuterà **gli aventi diritto**	Il Paese versa in una grave crisi economica. La prima operazione del nuovo governo sarà raddoppiare il fondo di disoccupazione. Molti cittadini pensano che sia una decisione giusta, perché aiuterà **gli aventi diritto**	Il Paese versa in una grave crisi economica. La prima operazione del nuovo governo sarà raddoppiare il fondo di disoccupazione. Molti cittadini pensano che sia una decisione giusta, perché aiuterà **chi è senza lavoro**
Probabilmente tutti concordano sul fatto che le creazioni di Leonardo da Vinci siano qualcosa di straordinario. Infatti **le sue capacità** affascinano da sempre grandi e bambini	Probabilmente tutti concordano sul fatto che le macchine da volo di Leonardo da Vinci siano qualcosa di straordinario. Infatti, **le sue capacità** affascinano da sempre grandi e bambini	Probabilmente tutti concordano sul fatto che le macchine da volo di Leonardo da Vinci siano qualcosa di straordinario. **Le sue capacità ingegneristiche** affascinano da sempre grandi e bambini
La legislazione sugli incidenti sul lavoro è ancora piuttosto debole. Dopo il grave incidente nel suo negozio, Luigi ha dovuto chiudere **l'attività** e cercarsi un nuovo impiego	La legislazione sugli incidenti sul lavoro è ancora piuttosto debole. Dopo essersi tagliato tre dita con l’affettatrice del suo negozio, Luigi ha dovuto chiudere **l'attività** e cercarsi un nuovo impiego	La legislazione sugli incidenti sul lavoro è ancora piuttosto debole. Dopo essersi tagliato tre dita con l’affettatrice del suo negozio, Luigi ha dovuto chiudere **la salumeria** e cercarsi un nuovo impiego
L'infiammazione di Giorgio gli sta causando dolori fortissimi e i suoi cari iniziano a preoccuparsi. Prima che la situazione peggiori, la famiglia si aspetta che i medici decidano di fare **qualcosa**	L’appendicite di Giorgio gli sta causando dolori fortissimi e i suoi cari iniziano a preoccuparsi. Prima che la situazione peggiori, la famiglia si aspetta che i medici decidano di fare **qualcosa**	L’appendicite di Giorgio gli sta causando dolori fortissimi e i suoi cari iniziano a preoccuparsi. Prima che la situazione peggiori, la famiglia si aspetta che i medici decidano di **operarlo**
Negli ultimi mesi Giulio è stato un pessimo studente. I suoi genitori stanno cercando di capire quale possa essere la causa, ma sono molto preoccupati e non sanno cosa fare. Temono **conseguenze serie**	Negli ultimi mesi Giulio ha avuto l’insufficienza in tutte le materie. I suoi genitori stanno cercando di capire quale possa essere la causa, ma sono molto preoccupati e non sanno cosa fare. Temono **conseguenze serie**	Negli ultimi mesi Giulio ha avuto l’insufficienza in tutte le materie. I suoi genitori stanno cercando di capire quale possa essere la causa, ma sono molto preoccupati e non sanno cosa fare. Temono **una bocciatura**
Tra due mesi Stefano dovrà fare da testimone al matrimonio della sorella. Entra in un negozio ma non gli piace nulla. Prima o poi troverà **qualcosa** di suo gradimento	Tra due mesi Stefano dovrà fare da testimone al matrimonio della sorella. Entra in un negozio di abbigliamento maschile ma non gli piace nulla. Prima o poi troverà **qualcosa** di suo gradimento	Tra due mesi Stefano dovrà fare da testimone al matrimonio della sorella. Entra in un negozio di abbigliamento maschile ma non gli piace nulla. Prima o poi troverà **un abito** di suo gradimento
Mario è un collezionista. Nel 2010 andò a Verona in occasione di una nota fiera che gli aveva consigliato un suo amico, ma non trovò **nulla di interessante**	Mario è un collezionista di mobili antichi. Nel 2010 andò a Verona in occasione di una nota fiera che gli aveva consigliato un suo amico, ma non trovò **nulla di interessante**	Mario è un collezionista di mobili antichi. Nel 2010 andò a Verona in occasione di una nota fiera che gli aveva consigliato un suo amico, ma non trovò **mobili interessanti**
Lorenzo è a Venezia e vorrebbe un caffè. Vede che nel portafogli ha qualche moneta e decide di entrare in un bar. Ancora non sa che a Venezia il caffè costa **molto**	Lorenzo è a Venezia e vorrebbe un caffè. Vede che nel portafogli ha due euro e decide di entrare in un bar. Ancora non sa che a Venezia il caffè costa **molto**	Lorenzo è a Venezia e vorrebbe un caffè. Vede che nel portafogli ha due euro e decide di entrare in un bar. Ancora non sa che a Venezia il caffè costa **cinque euro**
Daniele ha vinto dei soldi alla lotteria ed è molto indeciso su come spenderli. Non sa se fare due settimane di vacanza o acquistare **qualcosa** di design	Daniele ha vinto cinquemila euro ed è molto indeciso su come spenderli. Non sa se fare due settimane di vacanza o acquistare **qualcosa** di design per il salotto	Daniele ha vinto cinquemila euro ed è molto indeciso su come spenderli. Non sa se fare due settimane di vacanza o acquistare **un tavolo** di design per il salotto
Dopo il divorzio, Carla ha attraversato un periodo molto critico in cui non si voleva bene. Ora ha finalmente **cambiato abitudini,** ha nuovi amici ed è di nuovo felice	Dopo il divorzio, Carla ha attraversato un periodo molto critico in cui ha iniziato a bere. Ora ha finalmente **cambiato abitudini**, ha nuovi amici ed è di nuovo felice	Dopo il divorzio, Carla ha attraversato un periodo molto critico in cui ha iniziato a bere. Ora ha finalmente **smesso di bere,** ha nuovi amici ed è di nuovo felice
Federico tra pochi giorni deve partire per una vacanza al mare, e approfitta dei saldi per comprare degli occhiali senza spendere troppo. Nei primi tre negozi però non trova **nulla di adatto**	Federico tra pochi giorni deve partire per una vacanza al mare, e approfitta dei saldi per comprare degli occhiali da sole a meno di cento euro. Nei primi tre negozi però non trova **nulla di adatto**	Federico tra pochi giorni deve partire per una vacanza, e approfitta dei saldi per comprare degli occhiali da sole a meno di cento euro. Nei primi tre negozi però non trova **nulla per quel budget**
Valeria ha deciso di spendere i soldi della sua paghetta settimanale ed entra in un negozio di abbigliamento. La maglia che vorrebbe però è **costosa**, non può acquistarla	Valeria ha deciso di spendere i venti euro della sua paghetta settimanale ed entra in un negozio di abbigliamento. La maglia che vorrebbe però è **costosa**, non può acquistarla	Valeria ha deciso di spendere i venti euro della sua paghetta settimanale ed entra in un negozio di abbigliamento. La maglia che vorrebbe però **costa cinquanta euro**, non può acquistarla
Claudia vuole fare una torta di mele e la ricetta dice che le servono molte uova. Prima di iniziare però deve andare al supermercato perché in frigo ne ha **poche**	Claudia vuole fare una torta di mele e la ricetta dice che le servono otto uova. Prima di iniziare però deve andare al supermercato perché in frigo ne ha **poche**	Claudia vuole fare una torta di mele e la ricetta dice che le servono otto uova. Prima di iniziare però deve andare al supermercato perché in frigo ne ha **cinque**
Alla conferenza stampa tenuta dal Ministro della Salute hanno partecipato molti giornalisti. Per l’occasione è stata condotta un’indagine sull’uso di supporti elettronici ed è emerso che **solo un giornalista su dieci** prendeva appunti su carta	Alla conferenza stampa hanno partecipato cinquanta giornalisti. Per l’occasione è stata condotta un’indagine sull’uso di supporti elettronici ed è emerso che **solo un giornalista su dieci** prendeva appunti su carta	Alla conferenza stampa hanno partecipato cinquanta giornalisti. Per l’occasione è stata condotta un’indagine sull’uso di supporti elettronici ed è emerso che **solo cinque giornalisti** prendevano appunti su carta
All’esame di storia contemporanea Lucia ha preso un voto mediocre. Per alzare la sua media, puntava a **un voto più alto,** ma la laurea è vicina e dovrà accontentarsi	All’esame di storia contemporanea Lucia ha preso venticinque. Per alzare la sua media, puntava a **un voto più alto**, ma la laurea è vicina e dovrà accontentarsi	All’esame di storia contemporanea Lucia ha preso venticinque. Per alzare la sua media, puntava a **un trenta**, ma la laurea è vicina e dovrà accontentarsi
Negli anni Quaranta in Italia era normale sposarsi anche da giovanissimi per mettere su famiglia. Oggi la situazione è cambiata molto e tendenzialmente ci si sposa **più tardi**	Negli anni Quaranta in Italia era normale sposarsi anche prima dei diciotto anni per mettere su famiglia. Oggi la situazione è cambiata molto e tendenzialmente ci si sposa **più tardi**	Negli anni Quaranta in Italia era normale sposarsi anche prima dei diciotto anni per mettere su famiglia. Oggi la situazione è cambiata molto e tendenzialmente ci si sposa **dopo i venticinque anni**
Il liceo artistico di Novara ha vinto un bando e ha ricevuto un finanziamento di qualche migliaio di euro. **Buona parte dei fondi** verrà spesa per l’acquisto di nuovi computer	Il liceo artistico di Novara ha vinto un bando e ha ricevuto un finanziamento di novemila euro. **Buona parte dei fondi** verrà spesa per l’acquisto di nuovi computer	Il liceo artistico di Novara ha vinto un bando e ha ricevuto un finanziamento di novemila euro. **Seimila euro** verranno spesi per l’acquisto di nuovi computer
Per la prossima settimana, Alessio aveva pianificato due giorni da sogno con la sua fidanzata. Purtroppo però non si ricordava che avrà un’importante riunione di lavoro, e dovrà rimandare **i suoi piani**	Per la prossima settimana, Alessio aveva pianificato due giorni a Capri con la sua fidanzata. Purtroppo però non si ricordava che avrà un’importante riunione di lavoro, e dovrà rimandare **i suoi piani**	Per la prossima settimana, Alessio aveva pianificato due giorni a Capri con la sua fidanzata. Purtroppo però non si ricordava che avrà un’importante riunione di lavoro, e dovrà rimandare **il viaggio**
Da vent’anni a questa parte l’Europa investe molto nelle tecnologie del futuro, cercando di restare un continente competitivo nel mercato globale. Molti finanziamenti alla ricerca infatti sono finalizzati alla creazione di automobili **innovative**	Da vent’anni a questa parte l’Europa investe molto in tecnologie ecosostenibili, cercando di restare un continente economicamente competitivo nel mercato globale. Molti finanziamenti alla ricerca infatti sono finalizzati alla creazione di automobili **innovative**	Da vent’anni a questa parte l’Europa investe molto in tecnologie ecosostenibili, cercando di restare un continente economicamente competitivo nel mercato globale. Molti finanziamenti alla ricerca infatti sono finalizzati alla creazione di automobili **elettriche**
Giulio assiste a un evento sportivo. Il giocatore di punta della sua squadra del cuore subisce un infortunio ed è costretto a uscire dal campo **pochi minuti prima della fine**	Giulio assiste a una partita di calcio. Il giocatore di punta della sua squadra del cuore subisce un infortunio ed è costretto a uscire dal campo **pochi minuti prima della fine**	Giulio assiste a una partita di calcio. Il giocatore di punta della sua squadra del cuore subisce un infortunio ed è costretto a uscire dal campo **all’ottantasettesimo minuto**
Il notebook di Sergio si è danneggiato e lui lo porta in assistenza. Lì gli dicono che il computer è ancora in garanzia e che può avere **un intervento** gratuitamente	Il notebook di Sergio ha lo schermo rotto e lui lo porta in assistenza. Lì gli dicono che il computer è ancora in garanzia e che può avere **un intervento** gratuitamente	Il notebook di Sergio ha lo schermo rotto e lui lo porta in assistenza. Lì gli dicono che il computer è ancora in garanzia e che può avere **la sostituzione dello schermo** gratuitamente
Giacomo ha comprato le luci dell’albero di Natale, ma per accenderle sono necessarie molte batterie. A casa ne ha **pochissime**, quindi chiede al figlio di acquistarne altre tornando dall’ufficio	Giacomo ha comprato le luci dell’albero di Natale, ma per accenderle sono necessarie sei batterie. A casa ne ha **pochissime**, quindi chiede al figlio di acquistarne altre tornando dall’ufficio	Giacomo ha comprato le luci dell’albero di Natale, ma per accenderle sono necessarie sei batterie. A casa ne ha **due**, quindi chiede al figlio di acquistarne altre tornando dall’ufficio
A Natale Sara e i suoi amici volevano giocare a tombola, ma si sono accorti che mancava qualche numero da estrarre. Dopo una lunga ricerca, ne sono stati ritrovati **solo alcuni**	A Natale Sara e i suoi amici volevano giocare a tombola, ma si sono accorti che mancavano cinque numeri da estrarre. Dopo una lunga ricerca, ne sono stati ritrovati **solo alcuni**	A Natale Sara e i suoi amici volevano giocare a tombola, ma si sono accorti che mancavano cinque numeri da estrarre. Dopo una lunga ricerca, ne sono stati ritrovati **solo tre**
Luca suona da quando è piccolo. Ha molto talento, ma ora che è adulto teme di deludere le aspettative di sua madre, la quale spera che **faccia carriera**	Luca suona il piano da quando è piccolo. Ha molto talento, ma ora che è adulto teme di deludere le aspettative di sua madre, la quale spera che **faccia carriera**	Luca suona il piano da quando è piccolo. Ha molto talento, ma ora che è adulto teme di deludere le aspettative di sua madre, la quale spera che **diventi un grande pianista**
Passando davanti a un bar Giulia si rende conto che vuole prendere qualcosa ma ha dimenticato il portafogli. Fortunatamente, ha **un po’ di soldi** in tasca e decide di entrare	Passando davanti a un bar Giulia si rende conto che vuole prendere un caffè, ma ha dimenticato il portafogli. Fortunatamente, ha **un po’ di soldi** in tasca e decide di entrare	Passando davanti a un bar Giulia si rende conto che vuole prendere un caffè, ma ha dimenticato il portafogli. Fortunatamente, ha **un euro** in tasca e decide di entrare
Dopo una lunga organizzazione, Gaia domani finalmente riuscirà a vedersi con Gino per una passeggiata. Apre l’armadio alla ricerca di **qualcosa di adatto** da indossare, ma non trova nulla	Dopo una lunga organizzazione, Gaia domani finalmente riuscirà a vedersi con Gino per una escursione in montagna. Apre l’armadio alla ricerca di **qualcosa di adatto** da indossare, ma non trova nulla	Dopo una lunga organizzazione, Gaia domani finalmente riuscirà a vedersi con Gino per una escursione in montagna. Apre l’armadio alla ricerca di **pantaloni da trekking**, ma non trova nulla

## Footnotes

1 The notion of *non-*bona fide *true* content is found in Lombardi Vallauri and Masia (2014) It refers to contents that are not objectively true nor previously agreed upon among participants. In the case of vagueness, we consider vagueness as leaving implicit potentially *non-*bona fide *true* content, when the vague expression is not super-true in the Supervaluationist sense, i.e., when there are possible precisifications of it for which the proposition which includes them would result as false.

2 In this, we follow Peirce’s definition of *vague* (1902:748): "A proposition is vague when there are possible states of things concerning which it is intrinsically uncertain whether, had they been contemplated by the speaker, he would have regarded them as excluded or allowed by the proposition. By intrinsically uncertain we mean not uncertain in consequence of any ignorance of the interpreter, but because the speaker's habits of language were indeterminate.".

3 Further examples will be discussed in "Discussion" section.

4 For the present work, we specifically focus on the linguistic material employed in persuasive texts, but it is widely acknowledged that the persuasive potential of a textual product results from the combination and interplay of several multimodal variables, such as choice of images and music, which play a determinant role in conveying (implicit) messages and impacting in the receivers’ minds (Ansani et al. 2020; Ansani et al. 2019; Lombardi Vallauri 2019a, b).

5 Examples of Italian political speeches are taken from a corpus of 1500 Italian political speeches (from 1946 to the present day) which is planned to be collected, annotated for implicit strategies and made available online by 2023 within a Research Project IMPAQTS: Implicit Manipulation in Politics—Quantitatively Assessing the Tendentiousness of Speeches" (funded by the Italian Government as the Project of Relevant National Interest 2017, n. 2017STJCE9—https://impaqts.it/).

6 This definition should be intended in opposition to “implicitness of responsibility”, represented by other forms of implicit language such as presuppositions and topics (Lombardi Vallauri [Bibr CR40][Bibr CR43]).

7 In particular, this study shows that, since speakers implicating false contents are perceived as less committed to their truth as compared to speakers asserting or presupposing them, implicatures shield speakers from reputational risks to a greater extent.

8 In case the context has some role after all, non-precising context should, if anything, trigger longer processing as compared to precising, priming context. As a consequence, the outcome of the comparison between the three conditions we have considered (as we will see: precision takes more time than vagueness) should not be affected by not also measuring a condition D, where precise expression processing times could only be even longer than in condition C.

9 To be on safer side, an anti-ballot box stuffing was employed in order to avoid multiple participations from the same device.

10 As the Gamma distribution does not allow negative values, the parameterized RT were normalized between 0 and 1.

11 A similar GLMM model was run with not-parameterized RTs (Gamma distribution, Identity link function) with overlapping results.
